# Supplementary material for: Integrase-RNA interactions underscore the critical role of integrase in HIV-1 virion morphogenesis
Source: eLife. 2020 Sep 22;9:e54311. doi: 10.7554/eLife.54311 (PMC7671690; doi:10.7554/eLife.54311)
Supplement: Supplementary file 1. [file elife-54311-supp1.docx]

| **IN mutant** | **IN signal (%)** | **SD** | **IN mutant** | **IN signal (%)** | **SD** |
| --- | --- | --- | --- | --- | --- |
| WT | 100 | N/A | K186E | 63.2 | 16.4 |
| H12N | ND | N/A | R187A | 34.4 | 24.5 |
| K14A | 40.5 | 10.3 | K188E | 45.8 | 26.3 |
| N18I | 16.7 | 3.1 | R199A | 41.1 | 19.2 |
| K34A | 32.6 | 6.9 | Q214L/Q216L | 31.7 | 6.3 |
| E87A | 31.4 | \| 16.1 \| \| --- \| | K215A/K219A | 47.7 | 20.4 |
| E96A | 38.4 | 31.2 | R228A | 50.2 | 26.4 |
| Y99A | 22.0 | 10.3 | K236E | 37.4 | 14.0 |
| K103E | 6.8 | 2.3 | L241A | 41.4 | 12.4 |
| W108R | 2.7 | 0.3 | L242A | 13.7 | 3.5 |
| V165A | 55.6 | 32.8 | K258A | 36.7 | 19.7 |
| F185K | 2.5 | 1.6 | V260E | 6.0 | 4.0 |
| K186A | 69.4 | 27.9 | R262A/R263A | 52.9 | 7.0 |
