# Supplementary material for: Integrase-RNA interactions underscore the critical role of integrase in HIV-1 virion morphogenesis
Source: eLife. 2020 Sep 22;9:e54311. doi: 10.7554/eLife.54311 (PMC7671690; doi:10.7554/eLife.54311)
Supplement: Supplementary file 2. [file elife-54311-supp2.docx]

| **IN mutant** | **Multimeric species** | **Reference** |
| --- | --- | --- |
| H12N | Dimer or dimer/monomer mixture | Hare et al., *PLoS Pathog*, 2009 |
| K14A | Dimer | McKee et. al., *J Biol Chem*, 2008 |
| E87A | Dimer | Kessl et al., *Mol Pharmacol*, 2009 |
| F185K | Dimer | Pandey et. al., Biochem, 2011 |
| K186A | Dimer | McKee et. al., *J Biol Chem*, 2008; Houwer et. al., *J Biol Chem*, 2012 |
| K186E | Dimer or dimer/monomer mixture | Hare et al., *PLoS Pathog, 2009* |
| R187A | Dimer | McKee et. al., *J Biol Chem*, 2008; Houwer et. al., *J Biol Chem*, 2012 |
| L241A | Dimer | Luztke and Plasterk, *J Vir*, 1998 |
| L242A | Dimer/tetramer mixture, shifted to dimer | Luztke and Plasterk, *J Vir*, 1998 |
| R269A/K273A | Tetramers and monomers | Kessl et. al., *Cell*, 2016 |
